# Necrotizing Enterocolitis in Children with Congenital Heart Disease: A Literature Review

**DOI:** 10.1007/s00246-021-02691-1

**Published:** 2021-09-12

**Authors:** Sean T. Kelleher, Colin J. McMahon, Adam James

**Affiliations:** Department of Paediatric Cardiology, Children’s Health Ireland at Crumlin, Dublin 12, Ireland

**Keywords:** Congenital heart disease, Necrotising enterocolitis, NEC, Ductal dependent, Neonate, Microbiome

## Abstract

Infants with congenital heart disease (CHD) are at an increased risk of developing necrotising enterocolitis (NEC), a serious inflammatory intestinal condition classically associated with prematurity. CHD not only increases the risk of NEC in preterm infants but is one of the most commonly implicated risk factors in term infants. Existing knowledge on the topic is limited largely to retrospective studies. This review acts to consolidate existing knowledge on the topic in terms of disease incidence, pathophysiology, risk factors, outcomes and the complex relationship between NEC and enteral feeds. Potential preventative strategies, novel biomarkers for NEC in this population, and the role of the intestinal microbiome are all explored. Numerous challenges exist in the study of this complex multifactorial disease which arise from the heterogeneity of the affected population and its relative scarcity. Nevertheless, its high related morbidity and mortality warrant renewed interest in identifying those infants most at risk and implementing strategies to reduce the incidence of NEC in infants with CHD.

## Introduction

Necrotising enterocolitis (NEC) is a potentially devastating intestinal condition of infancy associated with a high morbidity and mortality. It arises when an immature or compromised gastrointestinal tract allows for the mucosal barrier to be breached by bacteria, resulting in an inflammatory cascade which may lead to ischemia and perforation [1]. It is most commonly seen in preterm infants, although it is not unique to this population. Congenital heart disease (CHD) is a well-described risk factor for the development of NEC in both term and preterm neonates [2, 3]. Among infants of normal birth weight (> 2500 g), CHD is reported to be implicated in up to 18% of cases [4]. However, despite CHD being one of the most common risk factors for NEC in term infants, the pathophysiology remains incompletely understood.

The aim of this review it to focus on NEC as it relates to infants with CHD with regards to the risk factors, pathophysiological mechanisms and outcomes that are specific to this group. NEC related to prematurity may be considered a separate entity and for the purposes of this review an isolated patent ductus arteriosus (PDA) in a preterm infant is not considered a congenital heart defect. Due to the substantive body of research in relation to NEC and prematurity, some comparisons between the two groups have been made throughout. Some authors have sought to compare differences in clinical presentation between infants with CHD and NEC and those with prematurity. Furthermore, infants with both CHD and prematurity are at a considerably higher risk of developing NEC than with either risk factor alone. As a result, many of the referenced observational works include results of both term and preterm infants with CHD. Finally, efforts to translate established preventative strategies in the preterm population to infants with CHD are discussed.

This topic present a challenge as available data largely come from retrospective studies many of which are from single centers [2, 5, 6]. The cohorts described may be heterogenous in terms of gestational age, birth weight and cardiac diagnoses. Furthermore, analysis requires controlling for multiple pre-operative and post-operative risk factors including co-morbidities, hemodynamic instability, cardiopulmonary bypass surgery and mode of enteral feeding [2, 7].

## Incidence

Incidence rates of NEC in infants with CHD have been reported between 3 and 5% [2, 5, 8, 9]. Much of the published literature comes from retrospective analysis of intensive care or peri-operative patients with the reported incidence reflecting more complex CHD. Incidence increases with decreasing gestational age [10]. It appears highest in hypoplastic left heart syndrome (HLHS) with rates between 6.1 and 9% [5, 11, 12].

## Pathophysiology

The pathophysiology of NEC in CHD is likely multifactorial. The impact of circulatory changes both acute and chronic cannot be overstated. We address both hemodynamic and intrinsic gut factors that may predispose this group to NEC.

### Hemodynamic Factors

#### Impaired Mesenteric Blood Flow

The concept of the “diastolic steal phenomenon” suggests that anatomic lesions that disrupt systemic perfusion during diastole, for example those with a duct-dependent systemic circulation, lead to impaired mesenteric blood flow which predisposes infants to NEC [2]. Carlo et al. demonstrated this phenomenon in 2007. They conducted a gestational age-matched and diagnosis-matched case–control study investigating risk factors for NEC in term infants with congenital heart disease. They demonstrated persistent retrograde diastolic flow in the abdominal aorta in 47% of infants with congenital heart disease who developed NEC, compared with 15% in controls (OR 5.04, 95% CI 1.07–23.82, *p* < 0.05). This was the only variable to achieve statistical significance although there were trends toward early feeding and formula feeding in the NEC group. The combination of diastolic flow reversal and NEC was seen in a variety of diagnosis including left-sided obstructive lesions, total anomalous pulmonary venous return, truncus arteriosus and Ebstein’s anomaly. These patients either had a patent ductus arteriosus (PDA) or truncus arteriosus and therefore had potential for physiological shunting. However, there was no difference in the presence of PDA, or reliance on prostaglandin between the NEC group or controls, and just over half of the infants who developed NEC did not demonstrate reversal of diastolic flow in the abdominal aorta [13]. Therefore, this may be only one of several mechanisms implicated in the pathophysiology of NEC in infants with CHD. The authors also questioned whether pulmonary vascular resistance could be responsible for the presence or absence of diastolic run-off in patients with an otherwise similar anatomic lesion.

Small studies have trialed the use of splanchnic near infrared spectroscopy (NIRS) as a surrogate marker for impaired tissue oxygenation and assessed the relationship between low regional oximetry (rSO2) values and the development of NEC [14, 15]. One such study demonstrated a trend toward lower splanchnic rSO2 values in patients with congenital heart disease and proven NEC using NIRS. A similar study in preterm infants demonstrated lower cerebral and splanchnic rSO2 values in infants with complicated NEC [16]. The use of splanchnic NIRS is complicated by the mobility of the gut within the abdominal cavity resulting in variability of recordings and low reproducibility [14].

In infants with HLHS impaired diastolic mesenteric blood flow may occur both before and after first stage palliation. A small prospective observational trial in 2005 demonstrated abnormal superior mesenteric artery (SMA) perfusion (as measured by diastolic flow direction and arterial resistance) in infants with HLHS both immediately before and after Norwood Procedure with Modified Blalock Taussig Shunt (BTS) [17]. More recently, first stage palliation of HLHS with a Sano shunt (right ventricle to pulmonary artery conduit) as opposed to BTS has been proposed as a strategy to limit diastolic blood flow from the systemic-to-pulmonary circulation. In an ancillary observational study to the Pediatric Heart Network Single-Ventricle Reconstruction Trial in 2011, mesenteric flow patterns and gastrointestinal outcomes post Norwood procedure were assessed in patients who had been randomized to either Sano procedure or BTS shunt. Doppler assessments of celiac artery flow characteristics were collected at discharge. While indicators of resistance to perfusion were higher in the BTS group, there was no significant difference in the rates of NEC between the two, although numbers were small [18].

Splanchnic blood flow is increased in response to enteral feeding [19]. The above studies controlled for this by performing ultrasonographic assessments while fasting or nil by mouth. Cheung et al. in 2003, demonstrated that diastolic flow in the SMA increased post-prandially in a group of infants palliated with a systemic-to-pulmonary artery shunt but remained either absent or reversed. However, there are no studies to date assessing the relationship between NEC and mesenteric blood flow changes in relation to the introduction of feeds.

#### Low Cardiac Output State and Shock

An additional proposed circulatory mechanism is that episodic hypoperfusion to the gut results in mesenteric ischemia and damage to the mucosal barrier. McElhinney et al. showed that episodes of low cardiac output and shock were significantly associated with NEC with an odd ratio of 6.5 (95% CI, 1.8–23.5). They evaluated risk factors for NEC in 643 term and preterm infants with CHD in both a cohort and case–control study [2]. EI Hassan et al. showed that in infants with HLHS, those that developed NEC were more likely to have required inotropes for greater than 7 days (73.9% vs 47.7%) but they were unable to differentiate if this complication occurred before or after development of NEC [12]. This highlights the difficulty of assessing physiological states retrospectively due to lack of available information [6].

#### Abnormal Systemic Vasculature

Independent of diastolic run-off and low cardiac output, Miller et al. in 2014 hypothesized that infants with HLHS may have abnormal systemic vasculature that predisposes them to NEC. In a retrospective cohort study of infants with HLHS undergoing stage I palliation at a single institution showed that the abdominal aorta pulsatility index was lower in those infants who developed NEC on both pre-operative and post-operative echocardiograms independent of right ventricular function. The authors argue that this implied differences in the systemic and mesenteric vasculature between those who developed NEC and those who did not. They speculated that the reasons behind this may be multifactorial arising antenatally from a complex interplay between maternal, fetal, and placental factors [20]. This concept may warrant further investigation, and there is minimal histological evidence that may support it at the current time. This research applies to infants with HLHS with duct-dependent systemic circulation specifically and has not been looked at in other infants with CHD.

### Gut Factors

Impaired perfusion to the gut has been hypothesized to be the initial insult that causes damage to the mucosal barrier which in turn could be breached by bacteria leading to an inflammatory cascade [21]. Factors intrinsic to the gut that may play a role include; gastrointestinal dysbiosis, feed type and changes to the mucosal barrier itself.

#### Effect of the Microbiome

A link between an abnormal gut microbiota and the development of NEC is well established [21–23]. Once a vulnerable intestinal mucosal barrier is breached by opportunistic bacteria, an inflammatory cascade begins which may lead to local ischemia and perforation [24]. The identity of specific causative pathogens is complex and involves bacteria and fungi that can be pathogenic in some hosts and benign in others [22]. A diverse intestinal microbiome may be protective, and preterm infants who develop NEC have been shown to have low diversity of gut flora [25]. Furthermore, prolonged empiric antibiotic courses have been shown to increase the risk of NEC and death in this population [26]. Term infants with critical congenital heart disease requiring repair in the neonatal period have been shown to have an altered intestinal microbiome when compared to healthy controls, with decreased total bacteria, decreased Actinobacteria, Bacteroidetes, Enterobacteriaceae species, and increased Firmicutes species [27].

In infants with CHD, it has been hypothesized that factors including courses of broad-spectrum antibiotics, exposure to nosocomial pathogens, use of infant formula and in-dwelling feeding tubes may play a role in increasing pathogenic gut bacteria and decreasing protective species [24, 27, 28].

#### Feed Type

Breast milk has been shown to play a protective role against the development of NEC in premature infants. Pathophysiological mechanisms for this include the wide array of immunoglobulins, growth factors, human milk oligosaccharides, prebiotics, as well as increasing protective Bifidobacterial and Bacteroides species [21]. The relationship between enteral feeds and the development of NEC in infants with CHD is complex and is discussed further below. But, there is some promising evidence that breast milk may play a protective role in this population also [7]. This requires further study and prospective work on the topic is lacking.

The complexities involved in the care for infants with congenital heart disease including, reduced somatic growth, prolonged hospital admission and high caloric requirements may contribute to low rates of human milk feeding. [2, 7, 8, 12, 14].

#### Mucosal Barrier

The mucosal barrier in infants with CHD who develop NEC is not well described and could represent an area of further research. Research has shown that those who develop NEC have been shown to have impaired regulation of intestinal microcirculatory perfusion which appears to play a significant role in the development of NEC [29]. Impaired motility [30], impaired microcirculatory perfusion [31] and, on a molecular level, increased expression of toll-like receptor 4 (TLR4) with resultant pro-inflammatory cascades [21] are all factors in this population. Whether similar inflammatory cascades are present in infants with CHD remains to be elucidated.

## Risk Factors

There are several described risk factors for NEC in infants with CHD which are described below and listed in Table 1.

**Table 1:** Risk factors for NEC in CHD

Risk factors	Comment
Prematurity	Risk increases with decreasing gestation [10]
Low birth weight	Increased risk reported in some but not all studies [6, 12]
	Associated with poorer outcomes and mortality[12]
Low cardiac output	Episodes of low cardiac output/shock associated with OR 6.5 for NEC[2]
Specific diagnoses	Hypoplastic left heart syndrome (incidence 6.1–9%) [5, 11, 12]
	Duct-dependent lesions – incidence 5% [5]
	Truncus arteriosus and aortopulmonary window [2]
	Atrioventricular septal defect in VLBW population [1]
Prostaglandin (PGE2)	Longer duration of prostaglandin may be associated with increased risk [6]
	Higher doses may be associated with increased risk [2]
RACHS-1 Score	Higher score associated with increased risk [5]
Associated syndromes	Trisomy 21: association demonstrated in VLBW infants [1]
Red Blood Cell Transfusion	Higher rates noted in infants who developed NEC. Causality not established[32]

### Prematurity

Prematurity is unsurprisingly correlated with the development of NEC in patients with CHD [2, 3, 8, 9]. Motta et al. in a retrospective cohort of over 4,000 infants with gestational ages at birth between 23 weeks and 34 + 6 weeks, demonstrated that infants with severe CHD (cyanotic lesions, left-sided obstructive lesions and congestive heart failure) had an increased risk of NEC with a relative risk of 3.72 (95% CI 1.37–10.1)[3]. Natarajan et al. in a comparative study on outcomes of late-preterm infants (34 weeks to 36 6/7 weeks) with CHD with term counterpart matched for cardiac diagnosis showed that even slightly lower gestational age puts infants at significantly higher risk for worse outcomes including NEC (23% vs 1.7%). These infants were also of significantly lower mean birth weight (2335 vs 3173 g) [8].

At lower gestations, lesions that might not significantly increase the risk of NEC in term infants, may have an impact. Bain et al. in a prospective study of very low birth weight infants, showed that the presence of atrial or ventricular septal defects was associated with an increased risk of NEC (OR 1.26, 95% CI 1.06–1.49) [33].

### Low Birth Weight

In a cohort of very low birth weight (< 1500 g) infants both with and without CHD, the presence of CHD has been reliably shown to significantly increase the risk of NEC (13% vs 9%) [1]. Among infants with CHD who develop NEC statistically significant lower birth weights have been reported in some but not all studies [6]. In a large multi-center cohort of patients with HLHS, ElHassan et al. did not show an increased risk of NEC, but outcomes were worse with low birth weight (< 2,500 g) [12]. The issue of low birth weight as a contributing risk factor for NEC in CHD is complicated by potential associated feeding issues and an adverse intra-uterine environment [34]. It may delay definitive surgical management, possibly leading to a more unbalanced and hemodynamically compromised circulation for a longer period. Furthermore, it is associated with higher all-cause mortality [2, 3, 5, 8, 12].

### Diagnoses Associated with NEC

McElhinney et al. showed that specific diagnoses including hypoplastic left heart syndrome (HLHS), truncus arteriosus and aortopulmonary window were independently associated with NEC [2]. The association with HLHS and NEC has been reliably replicated with rates of 6.1–9% reported [5, 12]. Single-ventricle defects made up 40% of all NEC cases in the McElhinney cohort [2].

Infants with a ductal dependent circulation reliant on prostaglandin (PGE) are postulated to have lower diastolic gut perfusion with the potential to lead to ischemia and NEC [6]. Lau et al. showed an incidence of NEC among patients with duct-dependent lesions of 5%, (*n* = 33/653), and patients with duct-dependent lesions accounted for 54% of their total NEC cases. However, they noted that most of these patients developed NEC after surgery (i.e., while no longer on PGE) [5]. Becker et al. in a large retrospective cohort study of 6710 infants showed a relatively low incidence (0.3%) of NEC while receiving PGE, of whom the majority (57%) were born < 37 weeks. Single-ventricle physiology was again highlighted as a significant risk in this cohort (OR 2.82, 95% CI 1.23–6.49). It did however find that those who developed NEC on average received PGE for a longer duration (26 vs 1 day) [6]. This link between duration of PGE therapy and NEC has been shown indirectly elsewhere, with prolonged PGE exposure in late-preterm infants felt to be one of several contributing factors to higher rates of NEC in these infants when compared to their term counterparts [8]. Interestingly, McElhinney et al. also demonstrated a higher risk of NEC with increasing doses of prostaglandin [2].

### Risk Scores

Lau et al. also showed that the use of a pre-operative risk assessment score (Risk Adjustment for Congenital Heart Surgery-1 score) can be helpful in predicting infants who will develop NEC based on the complexity of their diagnosis. They showed that a higher RACHS-1 score increased the risk of NEC, and that the infants with lower scores who developed NEC tended to have an additional risk factor such as prematurity [5].

### Trisomy 21, NEC and CHD

Infants with trisomy 21 have a high burden of disease within the first year of life, with rates of congenital heart disease of 51% and mortality of 9% [35]. The reported incidence of NEC in neonates with trisomy 21 was reported at 6.6% in those admitted to hospital by one study [36]. The incidence of structural anomalies of the gastrointestinal system is higher [37]. Trisomy 21 is also associated with immune dysregulation, which has been shown to be significant, involving both innate and adaptive immune systems, putting these patients at increased risk of infection and sepsis [38]. Despite the independent links between trisomy 21 and both NEC and CHD, it is rarely described as an independent risk factor for NEC in infants with CHD.

Fisher et al. in a large prospective cohort of very low birth weight (VLBW) infants (< 1500 g), compared rates of NEC in infants with CHD with that of those without CHD. They found in this VLBW population the single cardiac diagnosis associated with the highest statistically significant risk of NEC was atrioventricular septal defect (AVSD). AVSD has a strong association with trisomy 21 which was present in 55% of patients with AVSD. Patients with trisomy 21 without AVSD had an independently higher rate of NEC with a rate of 10.3% (AOR 1.59 95% CI 1.22 to 2.07). Patients with both trisomy 21 and AVSD had a NEC rate of 17.2% (AOR 2.09, 95% CI 1.22 to 3.56). AVSDs confer multiple physiological effects, including left to right shunting, valvular dysfunction, and cyanosis [1]. Boghossian et al. also showed that among VLBW infants with trisomy 21, the risk of NEC almost doubled in the presence of CHD [39]. There is, therefore, a paucity of research on the risk of NEC in term, normal birth weight infants with trisomy 21 and CHD.

### Red Cell Transfusion

Infants requiring surgery for CHD may require multiple red blood cell transfusions (RBCTs). A temporal relationship between the administration of RBCTs in premature infants and the subsequent development of NEC has been noted in several retrospective studies. However, causality has not been established and some argue that anemia itself is responsible [40, 41]. We found only one study which investigated a potential link in infants with CHD. In a 2014, retrospective case–control study of term infants in the post-operative period, a higher RBCT rate was noted in those that developed NEC than those that did not. However, the authors were unable to establish causality [32].

## Presentation

Typically, NEC presents with new onset feed intolerance, abdominal distension, and blood in stools. It can progress rapidly from subtle signs to systemic features include cardiorespiratory instability, sepsis and may lead to bowel perforation or death. The Modified Bell’s Criteria (Table 2) provide a standardized method of grading NEC according to clinical and radiographic features. Grade 1 includes cases of suspected NEC with mild systemic signs and early radiographic features which may be non-specific such as ileus. Grade 2 incorporates cases of radiologically confirmed NEC, with moderate systemic upset. Pathognomonic radiographic features include evidence of intra-mural air (pneumatosis intestinalis) or portal venous air. Grade 3 includes advanced systemic disease and shock with radiographic changes including ascites or perforation [21, 40].

**Table 2:** Modified bell’s criteria for NEC

Stage	Systemic signs	Intestinal signs	Radiological signs
I. Suspected			
A	Temperature instability, apnea, bradycardia	Elevated pregavage residuals, mild abdominal distension, occult blood in stool	Normal or mild ileus
B	Same as IA	Same as IA, plus gross blood in stool	Same as IA
II. Definite			
A: Mildly ill	Same as IA	Same as I, plus absent bowel sounds abdominal tenderness	Ileus, pneumatosis intestinalis
B: Moderately ill	Same as I, plus mild metabolic acidosis, mild thrombocytopenia	Same as I, plus absent bowel sounds, definite abdominal tenderness, abdominal cellulitis, right lower quadrant mass	Same as IIA, plus portal venous gas, with or without ascites
III. Advanced			
A: Severely ill, bowel intact	Same as IIB, plus hypotension, bradycardia, respiratory acidosis, respiratory acidosis, disseminated intravascular coagulation, neutropenia	Same as I and II, plus signs of generalized peritonitis, marked tenderness and distension of abdomen	Same as IIB, plus definite ascites
B: Severely ill: bowel perforated	Same as IIIA	Same as IIIA	Same as IIB plus pneumoperitoneum

*NEC* Necrotising enterocolitis – table compiled from references [21, 40]

NEC occurring in infants with CHD has different disease characteristics and presentation to that classically described in preterm infants. Steurer et al. showed, in a retrospective cohort study of over 2.9 million live births, that for every gestational age at birth, infants with critical congenital heart disease (who represented 0.23% of the total) had a higher incidence of NEC than counterparts born at the same gestation [10].

In single-center retrospective studies, infants with CHD who develop NEC are typically of higher gestational age and birth weight than those with classical preterm NEC. This was demonstrated by Siano et al., in a meta-analysis from 2019 [9, 42, 43]. Infants with CHD required surgery for NEC in 31% which was lower than non-CHD infants which were quoted at 66% in this meta-analysis [9]. All-cause mortality rates were higher in infants with cardiogenic NEC (38% vs 27%) [9].

Bubberman et al. reported that infants with CHD had more frequent involvement of the colon which they hypothesized was the result of ischemic injury in watershed zones to which the colon is more susceptible, and they argued this supported the concept of the “diastolic steal phenomenon”[44]. A study by Cozzi et al. did not corroborate these findings, with the primary site of involvement being the small intestine in infants with CHD and those without (33% vs 31%) [43].

Median post-natal age at diagnosis for cardiogenic NEC appears to be approximately 22–25 days[5, 6]. Becker et al. showed that premature infants with ductal dependent disease were diagnosed with NEC at a higher median post-natal age compared with term infants with similar conditions (22 [range 16–36] vs. 10 [range 4, 11] days, *p* = 0.01) [6]. Furthermore, Bubberman et al. showed that term infants with CHD were more likely to develop NEC at an earlier post-natal age than premature infants with no CHD (4 [range 2–24] vs. 11 [range 4–41] days *p* < 0.001)[44].

Pickard et al. described lower rates of secondary outcomes such as perforation, need for re-operation, strictures and need for stoma in infants with CHD and NEC vs those with NEC alone. However, the authors considered infants with an isolated moderate to large PDA as a congenital heart defect, while most consider this a separate entity. This makes interpreting the results difficult. Analysis of the infants in this group who had CHD other than an isolated PDA, did not reveal statistically significant results with regard to these secondary outcomes; however, it is worth mentioning no infants in this group developed strictures or short bowel syndrome indicating these outcomes may be less common [42].

## The Relationship Between Enteral Feeding, NEC and CHD

### Feeding in the Pre-operative Period

The issue of whether pre-operative enteral feeds are safe while receiving PGE remains controversial. Approximately 48% of NEC in CHD develops pre-operatively [9]. There is often hesitancy in feeding infants with CHD in the pre-operative period because of the perceived risk of NEC and adverse outcomes [45]. There is little convincing evidence that pre-operative feeds increase rates of NEC[11, 45, 46].

Much of our understanding in CHD comes from retrospective studies [11, 47]. Becker et al. showed a non-significant trend toward increased risk of NEC while enterally fed and receiving PGE (1.2/1000 vs 0.4/1000 infant days, *p* = 0.27) [6]. However, a meta-analysis did not show a significant difference in rates of NEC in those that were enterally fed on PGE versus those that were not, but the quality of the evidence was deemed very low [45]. Selection bias is an issue, as patients deemed high risk may not be enterally fed. In a retrospective cohort study of term infants with HLHS who were fed either trophic feeds (20–30 ml/kg/day) or kept nil by mouth before Norwood palliation, trophic feeds appeared safe, and resulted in a shorter duration of mechanical ventilation with a trend toward earlier post-operative feeding tolerance [48]. However, larger feed volumes (> 100 ml/kg/day) in the pre-operative period for infants with complex CHD, were associated with an increased risk of NEC in one study [7].

A 2016 survey of 46 units caring for infants with CHD showed wide variation in practice, with 65% allowing single-ventricle patients to feed prior to stage-one palliation procedures. This survey did not assess feed type or volume but rather feeding route. The majority (90%) allowed oral feeding where possible, and 63% allowed breast feeding. Among those that did not allow pre-operative feeds, the most commonly cited concern was NEC (100% of respondents), while 56% cited use of prostaglandin as an additional concern [49].

In Sweden, pre-operative enteral feeds are provided as routine. Noerdenström et al. retrospectively reviewed 458 infants with critical CHD (79% of whom received PGE) at their institution and showed that 97% received significant enteral feeds before cardiac surgery, with 88% (at least partially) receiving breast milk. They demonstrated low rates of NEC (*n* = 4, 0.9%), the majority (*n* = 3, 75%) of which developed in the post-operative period, with the remaining patient (*n* = 1, 25%), developing NEC on day 1, prior to feeding or surgery. The authors argue that this provides evidence that pre-operative enteral feeds are safe and suggest that breast milk may be protective in this cohort. However, this is an observational inference. Low rates of NEC, the authors acknowledge, may be in part attributable to fewer episodes of pre-operative hemodynamic instability. [50].

### Introduction of Feeds in the Post-operative Period

The post-operative period for cardiac procedures (both open heart surgery and cardiac catheterisation) is a high-risk period for the development of NEC [51]. The reasons for which are likely to be multifactorial including pre-operative morbidity, cardiopulmonary bypass, post-operative hemodynamic instability as well as enteral feed re-introduction. A recent systematic review showed just over half (52%) of cases of cardiogenic NEC occur in the post-operative period [9]. The incidence of post-operative NEC in a UK multi-center review of surgical morbidity was 2.4%. However, this study included all pediatric patients undergoing cardiac surgery under the age of 17, with infants and neonates accounting for 53% of the total. This significance of this result is therefore difficult to interpret [52].

The role that enteral feeds play in post-operative NEC is challenging to assess. Iannucci et al. in 2013, noted that 60% of NEC cases at their institution occurred in the post-operative period following the introduction of enteral feeds. They conducted a matched case–control analysis of enteral feeding patterns in those infants who developed NEC and those who did not and could find any differences between the two groups with regard to caloric density of feeds, formula type, post-operative day of feed initiation or feeding velocity. Interestingly, post-operative feeds had not yet been introduced in 27% of infants at time of development of NEC [53]. This 8-year review acknowledged that feed advancement strategies were not protocol driven and clinical practice may have varied over the study period.

One of the few studies to assess an interventionist strategy in reducing the incidence of NEC in CHD, was performed by del Castillo et al. in 2010. This study retrospectively assessed the impact of the introduction of a post-operative enteral feed advancement protocol in patients with HLHS following stage-one palliative surgery. The slow introduction of either breast milk or hydrolyzed formula over an average of 7 days post-operatively was recommended. Utilizing this strategy, a statistically significant reduction in the rates of NEC from 27% to 6.5% was shown (*p* < 0.01) [54]. Of note, initial rates of NEC reported in this study are above those reported elsewhere.

## Preventative Strategies

Established strategies for prevention of NEC in preterm infants include exclusive use of breast milk [21, 55] and administration of probiotics [56]. Efforts to assess their usefulness in infants with CHD are discussed below as well as novel biomarkers for NEC in CHD.

### Breast Milk and CHD

In a retrospective cohort study of 546 infants with complex CHD, Cognata et al. in 2020, showed a pre-operative incidence of NEC of 3.3% with a median age of onset at 8.5 days. While controlling for multiple factors, they found that an exclusive unfortified human milk diet was associated with a significant reduction in the risk of pre-operative NEC Bell Stage I–III (OR 0.17, 95% CI 0.04–0.84) [7]. Further evidence is required to substantiate this protective effect.

### Use of Probiotics in CHD

There are several small pilot studies or retrospective cohorts on probiotic use in CHD. In terms of their impact on the fecal microbiota, probiotic Bifidobacterium Longum ssp. was not shown to alter the fecal microbiota of infants with CHD requiring surgery within the first few weeks of life in a small RCT (*n* = 16) [27]. Whereas administration of Bifidobacterium Breve appeared to improve the intestinal environment (reduced Enterobacteriaceae and Pseudomonas) in another similar RCT with small numbers (*n* = 21) of patients undergoing surgery between 1 and 2 weeks of life and who received probiotics pre- and post-operatively. There was a non-statistically significant trend toward earlier PICU discharge and days to enteral feeding in the probiotic arm [28]. A trend toward improved outcomes with administration of probiotics was also shown by Dilli et al. in 2014, in a randomized controlled trial of 100 infants with cyanotic congenital heart disease receiving Bifidobacterium Lactis (plus inulin), with lower rates of culture proven sepsis (18% vs 4%, *p* = 0.03), NEC (10% vs 0%) and death (28% vs 10%, *p* = 0.04) [57]. A retrospective cohort study on the use of dual strain probiotics in infants with duct-dependent CHD, observed a reduction in the frequency of NEC in infants with arch obstruction when probiotics were administered (5.6 vs 0%, *p* = 0.048) [58].

### Predictive Strategies and Biomarker Use

Future preventative strategies may include the use of biomarkers to identify post-operative patients at risk of NEC. Fecal calprotectin is a neutrophil activation biomarker which can be elevated in inflammatory disease of the gastrointestinal system. A prospective pilot study of 30 patients with duct-dependent CHD in the post-operative period showed statistically significant higher levels of fecal calprotectin in those that developed post-operative NEC [59]. Circulating intestinal fatty acid binding protein is a biomarker for enterocyte injury. In an observational cohort study of 120 infants undergoing cardiopulmonary bypass, intestinal fatty acid binding protein was measured at 6 and 24-h post-surgery. Significantly higher levels were noted in patients who subsequently developed NEC [60].

## Outcomes

### Mortality

NEC appears to carry a high overall mortality rate in infants with CHD, with in hospital mortality rates of 19–26% quoted [2, 5]. However, discussion surrounding mortality in infants with CHD and NEC must consider the differences in definitions used.

Some authors quote cases inclusive of Bell’s Stage I-III, that is, including cases of suspected or presumed NEC, whereas others will exclude Bell’s Stage I cases. For example, Becker et al. quoted high overall mortality rates of 48% in their cohort of infants with duct-dependent lesions and NEC. This may reflect their exclusion of patients with suspected NEC. Of the patients that died in this study, 70% were preterm. The reported mortality in term infants was 14%. They reported their mortality rate of infants with duct-dependent heart disease who did not have NEC as 7%. Bubberman et al. reported a similar overall mortality rate of 39% in infants with CHD who develop NEC of severity Bell’s Stage II or greater. However, they reported their mortality rate as a consequence of NEC as 11% [44]. McElhinney et al. in a case–control study of infants with CHD, did not show significantly higher mortality in those with NEC versus those without (Bell’s Stage I–III). However, the authors acknowledge that all the deaths in patients with NEC were directly attributable to NEC. Furthermore, they did show that among patients with stage 3 disease there was a significantly greater risk of death [2].

Fisher et al. demonstrated that in VLBW infants with CHD, mortality was higher in those that developed NEC (55% vs 34%). Mortality from CHD and NEC together was higher than either disease alone [1]. The presence of congenital heart disease in an infant who develops NEC was associated with up to seven-fold increased risk for mortality compared to those without CHD in one case–control study. Patient demographics here were not matched for gestational age or birth weight and therefore may not be an accurate comparison [61]. A meta-analysis on the subject showed higher mortality rates in NEC if CHD is present with an odd ratio of 3.4 (95% CI 2.8–4.1)[9].

ElHassan et al. in a large multi-center cohort of 5720 infants with HLHS following Stage 1 palliation or heart transplant showed an overall rate of NEC (Stage I–III) of 6.1%. Interestingly, while on univariable analysis they showed a higher mortality rate in infants who developed NEC than those who did not (23.5 vs 13.9%, *p* < 0.001), neither medical nor surgical NEC was a significant predictor of mortality on multivariable analysis. While intuitively it would seem that NEC would lead to higher mortality rates in CHD, this is a heterogenous group of patients that makes retrospective comparisons of mortality complex.

### Secondary Outcomes

NEC has been shown to be associated with a longer duration of hospital stay, mechanical ventilation and parenteral nutrition [2, 12]. The medical management involves close observation, cessation of enteral feeding and broad-spectrum antibiotics (including anaerobic cover), for periods of up to 14 days. Surgical management of NEC entails either placement of an intra-abdominal drain or exploratory laparotomy with resection of diseased bowel if necessary. Surgical morbidity includes short gut syndrome, need for re-operation, strictures, requirement for stoma placement and post-operative infection [40, 42, 62]. Rates of surgical intervention for NEC in CHD are reported to be between 15% and 38% [2, 5, 12].

## Discussion

Much remains to be elucidated in the topic of NEC in infants with CHD, despite being one of the leading risk factors for NEC in the term population. It is a challenge to investigate due its relatively low prevalence and the heterogeneity of the population. All potential risk factors have yet to be identified, with our review suggesting that trisomy 21 is a potentially overlooked risk factor [1, 39].

What makes the mucosal barrier of infants with CHD susceptible to NEC is not clearly understood. Hypoperfusion appears to play a central role [13] and episodes of low cardiac output certainly increase the risk [2]. Infants with a duct-dependent circulation are at an increased risk, likely due to relative diastolic hypoperfusion of the mesentery while reliant on prostaglandin. It appears that longer time spent reliant on prostaglandin increases the risk of NEC [6]. Interestingly, it would appear many infants with duct-dependent circulation develop NEC after surgical correction [5, 6], suggesting that an initial ischemic insult may manifest later. This may be in response to the hemodynamic changes surrounding cardiopulmonary bypass. In infants with HLHS, impaired SMA perfusion may persist post-operatively. It is also possible that post-operative medications could play a role. Vasopressin in animal models has been shown to decrease splanchnic blood flow [63] and there is concern that this could result in an increase in NEC. Opioid medications decrease gut motility and affect feeding, but whether they increase NEC is not clear, there is some evidence from the preterm population that they are safe [64].

Fecal calprotectin and intestinal fatty acid binding protein may become useful laboratory biomarkers for NEC [59, 60]. Doppler ultrasonography of the mesenteric vessels and abdominal aortic blood flow in diastole may be increasingly used as a predictive strategy, which could increase vigilance among practitioners [13]. Near-infrared spectroscopy (NIRS) may be of clinical use in predicting which infants are at highest risk but larger studies are needed to assess its utility in infants with CHD [16, 65, 66].

With regards to the relationship with feeds and NEC, in the pre-operative period, several retrospective studies have shown that enteral feeding appears safe, but this has not been shown at higher volumes and with all diagnoses [7, 45, 48]. Further work is needed on infants with CHD who receive breast milk but it does appear to confer some protection against the development of NEC [7, 50, 54]. Likewise, post-operative feed advancement protocols tailored for use in CHD may improve outcomes for this vulnerable cohort of infants. Most of the existing knowledge on the microbiome in CHD comes from small studies, and this may be an exciting area of research in the future. Infants with CHD appear to have decreased diversity of gut flora [27]. Whether this leads to an increased risk of NEC and whether this could be targeted with specific probiotic therapy remains to be discovered.

## Conclusions

The pathophysiology of this complex disease in the context of CHD is not well understood.

Preventative strategies require further investigation in infants with CHD and are lagging behind those of preterm counterparts. Emerging areas of research include the use of biomarkers for NEC and the role of the intestinal microbiome.

## Data Availability

Not applicable.
